# P-993. Increased Reporting of RPR Seronegative Infants with Congenital Syphilis to the Chicago Department of Public Health, A Quality Improvement Project

**DOI:** 10.1093/ofid/ofae631.1183

**Published:** 2025-01-29

**Authors:** John Flores, Yeo Won Ahn, Nikki Kasal, Osasu Osayande, Amy Betz, Allison H Bartlett

**Affiliations:** University of Chicago Hospital, Chicago, Illinois; The University of Chicago, Chicago, Illinois; University of Chicago Pritzker School of Medicine, Chicago, Illinois; University of Chicago Medicine, Chicago, Illinois; University of Chicago Medicine, Chicago, Illinois; University of Chicago Comer Children's Hospital, Chicago, IL

## Abstract

**Background:**

We are in the midst of an epidemic of congenital syphilis (CS). Nationwide CS diagnosis and treatment recommendations provided by the Centers for Disease Control are dependent on the timing of maternal treatment, and laboratory and procedural results of the mother and infants. There are specific circumstances where infants with a non-reactive rapid plasma reagin (RPR-) should receive a 10-day course of treatment in the context of inadequate maternal treatment. Depending on the institutional reporting practices of CS to departments of public health, there may be scenarios where RPR-based reporting protocols will leave out this specific cohort.

**Figure:**
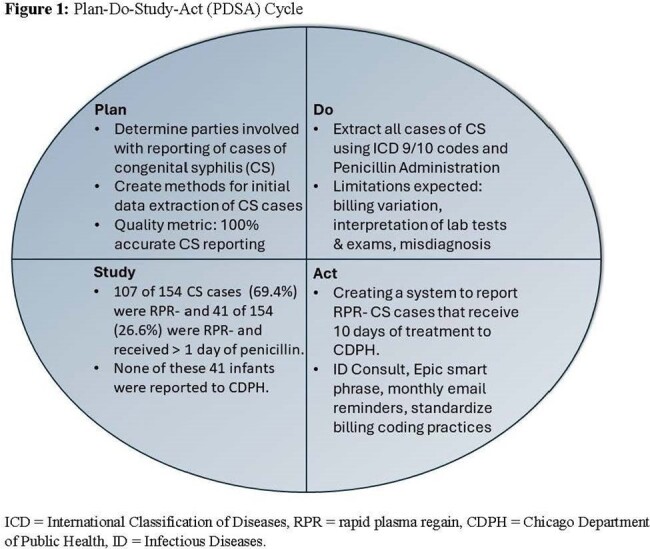

**Methods:**

Baseline data of Infants stratified by RPR status was collected through electronic medical record (EMR) review for those born at the University of Chicago Comer Children’s Hospital in Chicago, IL between January 2011 and December 2022, using billing codes & penicillin administration data. From September 1^st^, 2023, through March 31^st^, 2024, a quality improvement intervention was implemented that involved communicating with the hospital’s Pediatric Infectious Diseases specialists, Pediatric & Newborn Hospitalists, Neonatologists, Infection Prevention & Control, and Chicago Department of Public Health (CDPH). The intervention components included 1) formalize expectation to page Pediatric ID on-call team all infants with concern for maternal syphilis exposure, 2) create an Epic Smart Phrase to standardize data presentation for clinical and reporting purposes, 3) standardize ICD-10 diagnosis coding practices, and 4)email reminders monthly to teams involved.

**Figure:**
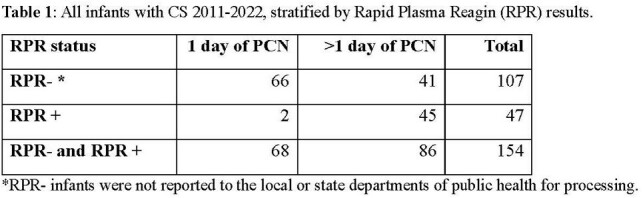

**Results:**

154 infants were identified who had a CDC case definition of CS and received penicillin administration therapy. Infants with CS from 2011-2022 showed 107/154 (69.4%) were RPR- and 41/154 (26.6%) were RPR- and received > 1 day of penicillin. At the end of the first PDSA cycle, a total of 22 infants were reported to CDPH with 36% (n=8) being RPR-.

**Figure:**
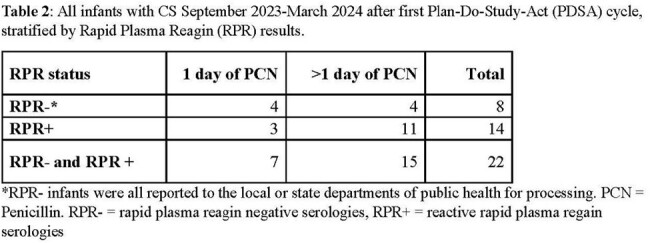

**Conclusion:**

Our Quality Improvement project identified a large percentage of infants diagnosed and treated with CS who were not reported to the CDPH with our previous RPR-positive – based reporting strategy. We implemented an intervention that successfully improved reporting of this specific type of patient.

**Figure:**
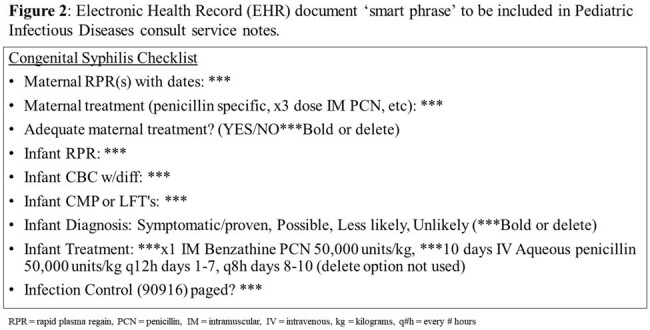

**Disclosures:**

**Allison H. Bartlett, MD, MS**, CVS/Caremark: Advisor/Consultant

